# Application of Fluorescence In Situ Hybridization (FISH) Technique for the Detection of Genetic Aberration in Medical Science

**DOI:** 10.7759/cureus.1325

**Published:** 2017-06-09

**Authors:** Zubair Ahmed Ratan, Sojib Bin Zaman, Varshil Mehta, Mohammad Faisal Haidere, Nusrat Jahan Runa, Nasrin Akter

**Affiliations:** 1 Department of Biomedical Engineering, Khulna University of Engineering and Technology, Bangladesh.; 2 Maternal and Child Health Division, International Centre for Diarrhoeal Disease Research, Bangladesh; 3 Department of Internal Medicine, MGM Medical College, Navi Mumbai, India; 4 Department of Soil, Water and Environment, University of Dhaka, Bangladesh.; 5 Department of Biochemistry, Gazi Medical College, Khulna, Bangladesh.; 6 Medicine, Yamagata University Faculty of Medicine, Japan

**Keywords:** fluorescence in situ hybridization, genetic anomalies, medical science

## Abstract

Fluorescence in situ hybridization (FISH) is a macromolecule recognition technique, which is considered as a new advent in the field of cytology. Initially, it was developed as a physical mapping tool to delineate genes within chromosomes. The accuracy and versatility of FISH were subsequently capitalized upon in biological and medical research. This visually appealing technique provides an intermediate degree of resolution between DNA analysis and chromosomal investigations. FISH consists of a hybridizing DNA probe, which can be labeled directly or indirectly. In the case of direct labeling, fluorescent nucleotides are used, while indirect labeling is incorporated with reporter molecules that are subsequently detected by fluorescent antibodies or other affinity molecules. FISH is applied to detect genetic abnormalities that include different characteristic gene fusions or the presence of an abnormal number of chromosomes in a cell or loss of a chromosomal region or a whole chromosome. It is also applied in different research applications, such as gene mapping or the identification of novel oncogenes. This article reviews the concept of FISH, its application, and its advantages in medical science.

## Introduction and background

The study of the genetics of cells and molecular biology has helped us to build up some ‘in situ hybridization’ techniques [1], through which congenital disorders or abnormalities can be detected to handle the cases more effectively and efficiently during the clinical practices [2]. The level of accuracy and specificity in these diagnoses play the pivotal role in treating, curing, preventing, and lessening the pain and agony of the patients as well as pave the way for further clinical developments in these fields of medical sciences [3]. 

The detection of specific gene sequences on the chromosome, or either its presence or absence, is the central concern of cytogenetic technique in diagnosing as well as enumerating a genetic disorder or abnormalities. Among the tools of cytogenetic techniques, a technique named fluorescence in situ hybridization (FISH) was developed in the early 1980's [4]. Applications of the FISH assay have been on the rise since the 1990's. Fluorescent deoxyribonucleic acid (DNA) probes, which are attached to the high degree of complementary parts of the chromosome, emit the colored signals. These signals could be grasped and visualized using fluorescent DNA probes, which further unveiled another way of detecting genetic abnormalities in medical science [5].

FISH is a less time-consuming technique in comparison to the conventional method of cytogenetic metaphase karyotype analysis [6] because, in the former, the detection technique involves processing with either fresh water or paraffin-embedded interphase nuclei without the need of culturing [7]. By using this technique, specific cytogenetic abnormalities, as well as a copy of aberrations numbers, can be enumerated and sketched. For example, chromosomal microdeletion, amplification, and subsequently, translocation can easily be detected through FISH. The improved diagnostic techniques have made the treatment easier and increased the life expectancy of people [8]. Therefore, FISH is becoming a more vital tool to detect and monitor the specific therapy with regards to the gene abnormalities; for example, the detection of the BCR/ALB1 translocation in chronic myeloid leukemia, human epidermal growth factor receptor 2 (HER2) augmentation in breast cancer, and anaplastic lymphoma kinase (ALK) rearrangement in adenocarcinoma [9-10]. The aim of this article is to review the concept of FISH, its growing need for application, and its advantages in medical science.

## Review

### Mechanism of FISH

The Basic Elements of FISH: DNA Probe and a Target Sequence

**Figure 1:**
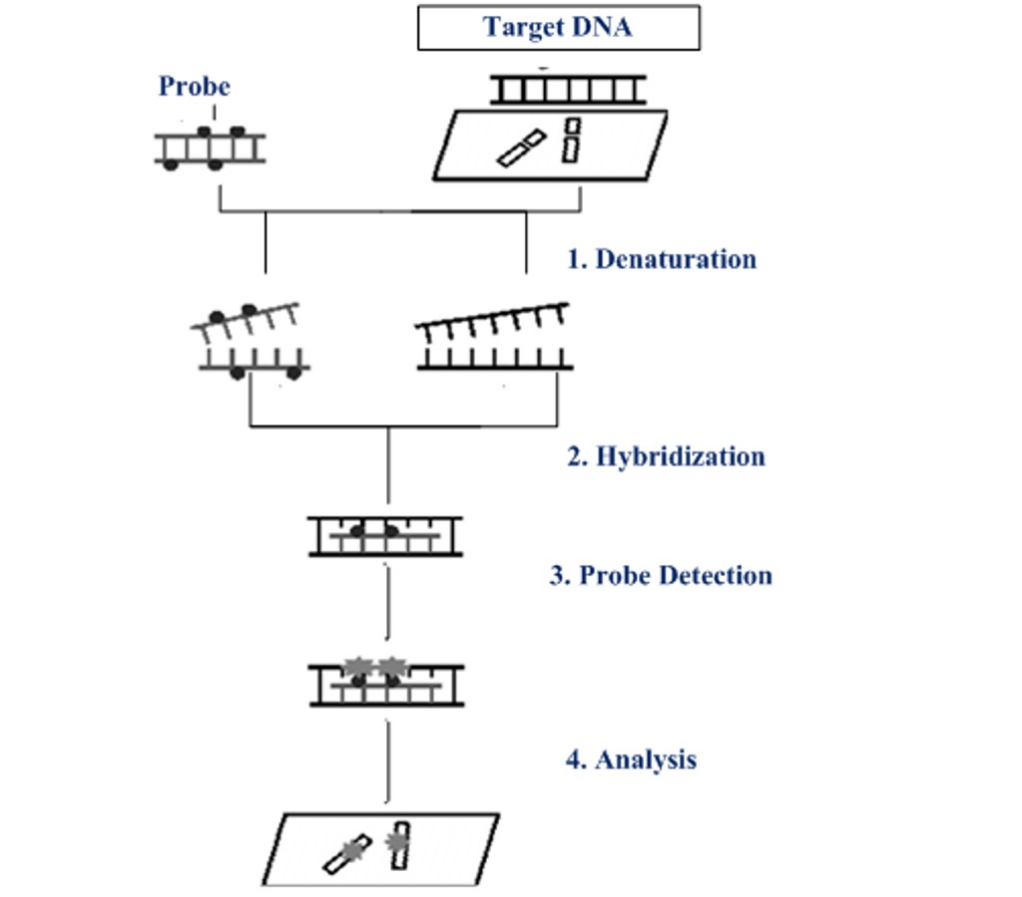
Basic steps of fluorescent in situ hybridization technique DNA: deoxyribonucleic acid

The first step is to prepare short sequences of single-stranded DNA that match a portion of the gene that we are looking for. These are called probes. The DNA probe is labeled in various ways, such as nick translation, random primed labeling, and PCR. Two labeling strategies are used: indirect labeling and direct labeling. In the case of direct labeling, probes are being labeled with nucleotides containing a fluorophore. In indirect labeling, it is the modified nucleotides that contain a hapten at which probes are being labeled. Then, the labeled probe and the target DNA are denatured. The annealing of complementary DNA sequences happens due to the combining consequences of denatured probe and target DNA. In the case of indirect labeling, an extra step is needed for visualization of the non-fluorescent hapten that uses an enzymatic or immunological detection system [2]. However, the selections of the FISH probe are dependent upon the diseases, anomalies, or anomalies under the field of interest [11]. Basic steps of the FISH procedure is depicted in Figure 1.

### Methodology

Search Strategy and Keywords

A literature search was done in PubMed, PubMed Central, and Google Scholar using strings of keywords as follows: fluorescence in situ hybridization, genetic anomalies and their correction, and application of FISH with dates ranging from January 1984 to December 2015. After completion exclusion of repetition was done, then articles were chosen purposively according to the inclusion and exclusion criteria. From total 3,024 articles, 57 articles were finally selected for review.

Inclusion and Exclusion Criteria

We included the articles from 1984 to 2015, which had full text in PDF format and were available in English. Articles without quantitative data and whose outcome evaluation methods were different were also excluded.

Data Extraction

**Figure 2:**
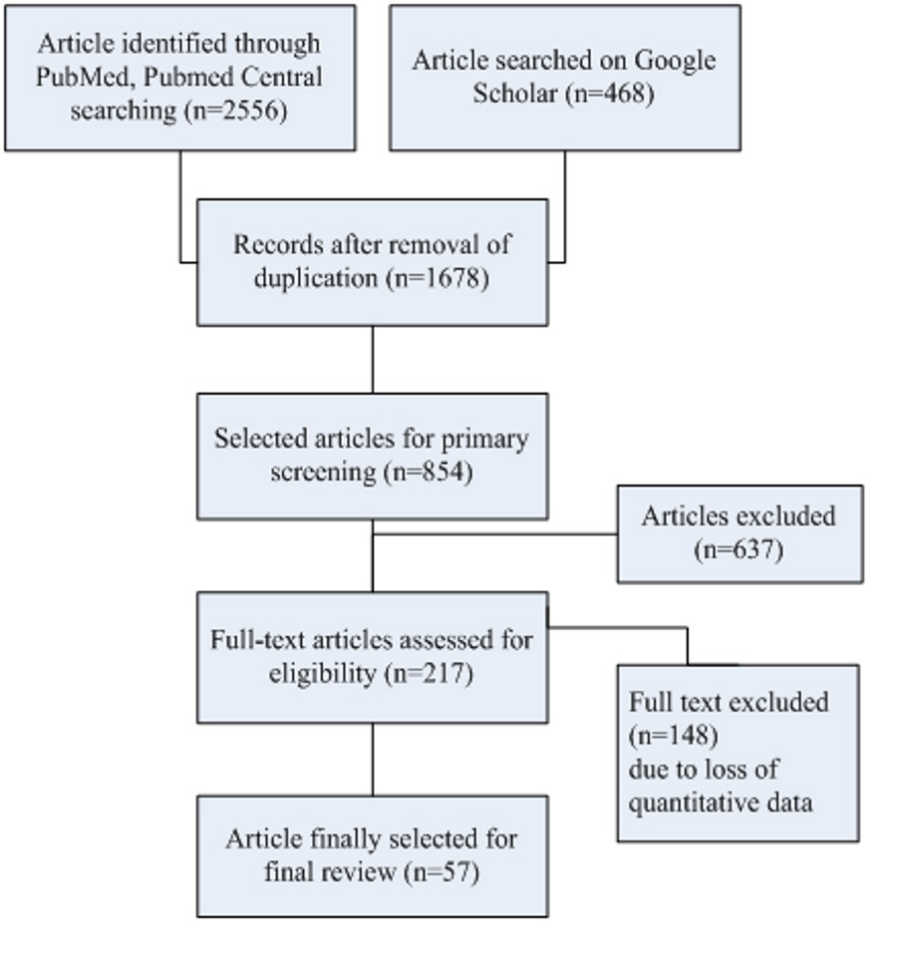
Flow chart of literature search

The total of 3,024 articles was found. After exclusion of repeated articles (1,346), 1,678 articles were left. From the 1,678 articles, 854 article were selected by screening the title and abstracts manually. Eight hundred and fifty-four articles were screened and 217 were selected for review after assessing the full text. Finally, 57 articles were selected after applying the exclusion criteria (Figure 2).

Reliability Assessment

Reliability was assessed in forms of the internal consistency, test-retest reliability and inter-rater reliability, and split-half reliability. Most of the articles assessed the internal consistency form of reliability, and in all articles, reliability was measured by Cronbach’s alpha having a level of ≥ 0.70.

### Results

Different types of FISH and their function are shown in Table 1.

**Table 1:** Findings of the Studies that Used the Fluorescence In Situ Hybridization (FISH) Technique CARD-FISH: catalyzed reporter deposition FISH; CAT-FISH: capture antibody targeted detection FISH; CB-FISH: cytochalasin B FISH; CO-FISH: cyotochrome orientation FISH; COD-FISH: chromosome orientation and direction FISH; D-FISH: dual color FISH; DBD-FISH: deoxyribonucleic acid breakage detection FISH; DNA: deoxyribonucleic acid; M-FISH: multiple spectral karyotyping FISH; ML-FISH: multilocus FISH; mRNA: messenger ribonucleic acid; PCC-FISH: premature chromosome condensation FISH; Q-FISH: quantitative FISH; QD-FISH: quantum dots FISH; RNA: ribonucleic acid; T-FISH: tissue FISH

Sr. No.	Different Types	Function	Name of the study	Author	Year
1	ACM-FISH	In sperm cell, structural and numerical chromosomal abnormalities can be detected	Integrating new tests of sperm genetic integrity into semen analysis: breakout group discussion	Perreault, et al. [12]	2003
2	armFISH is a 42-color M-FISH variant	Abnormalities at the chromosomal arms (p- and q-arms of all 24 human chromosomes; exception: p-arm of the Y and acrocentric chromosomes)	Arm-specific multicolor fluorescence in situ hybridization reveals widespread chromosomal instability in glioma cell lines	Sallinen, et al. [13]	2003
3	CARD-FISH	Amplification of signal which is obtained by peroxidase activity	Detection of activity among uncultured Actinobacteria in a drinking water reservoir	Nielsen, et al. [14]	2006
4	CAT-FISH	Expression of genes patterns in brain	Environment-specific expression of the immediate-early gene Arc in hippocampal neuronal ensembles.	Guzowski, et al. [15]	1999
5	CO-FISH	The orientation of tandem repeats in the centromeric regions of chromosomes	Strand-specific FISH reveals the orientation of chromosome 18 alphoid DNA.	Goodwin, et al. [16]	1993
6	CB-FISH	The cytological analysis of micronucleation and aneuploidy	Detection of mosaic chromosome 21 aneuploidy in vivo with the CB-FISH method.	Chen, et al. [17]	2000
7	COD-FISH	Quantification of gene copy number and the protein amount	A new method for detecting pericentric inversions using COD-FISH.	Bailey, et al. [18]	1996
8	Comet-FISH	DNA damage	Modification of the alkaline Comet assay to allow simultaneous evaluation of mitomycin C-induced DNA cross-link damage and repair of specific DNA sequences in RT4 cells.	McKenna, et al. [19]	2003
9	D-FISH	Detection of BCR/ABL fusion in chronic myeloid leukemia (CML)	A two color BCR-ABL probe that greatly reduces the false positive and false negative rates for fluorescence in situ hybridization in chronic myeloid leukemia	Grand, et al. [20]	1998
10	DBD-FISH	Any sites of DNA damage/breakage in the sample genome	Application of FISH to detect DNA damage. DNA breakage detection-FISH (DBD-FISH).	Fernandez, et al. [21]	2002
11	Fiber-FISH	Mapping of genes and chromosomal regions on fibers of chromatin or DNA	High-resolution DNA fiber-FISH for genomic DNA mapping and colour bar-coding of large genes.	Florijn, et al. [22]	1995
12	Flow-FISH	visualize and measure the length of telomere	Telomere length dynamics in human lymphocyte subpopulations measured by flow cytometry	Rufer, et al. [23]	1998
13	Fusion-Signal FISH	In peripheral blood and bone marrow 9;22 Philadelphia translocation is detected	Detection of a minimal residual disease state in chronic myelogenous leukemia patients using fluorescence in situ hybridization	Amiel, et al. [24]	1994
14	Harlequin-FISH	Cell cycle-controlled chromosome analysis in human lymphocytes	Detection of chromosome aberrations by FISH as a function of cell division cycle (harlequin-FISH)	Jordan, et al. [25]	1999
15	Immuno-FISH	Both DNA and proteins can be analyzed in the same sample	Association of transcriptionally silent genes with Ikaros complexes at centromeric heterochromatin	Brown, et al. [26]	1997
16	M-FISH	Facilitating the analysis of complex chromosomal rearrangement	Multicolor spectral karyotyping of human chromosomes	Schrock, et al. [27]	1996
17	ML-FISH	Identifying multiple microdeletion syndromes in patients	Simultaneous, multilocus FISH analysis for detection of microdeletions in the diagnostic evaluation of developmental delay and mental retardation	Ligon, et al. [28]	1997
18	PCC-FISH	Chromosome damage after irradiation	The prediction of human tumor radiosensitivity in situ: an approach using chromosome aberrations detected by fluorescence in situ hybridization	Brown, et al. [29]	1992
19	Q-FISH	Determining the repeated number of telomere on a specific chromosome	Short telomeres on human chromosome 17p	Martens, et al. [30]	1998
20	QD-FISH	Human metaphase chromosomes, human sperm cells, bacterial cells, and also to detect subcellular mRNA distribution in tissue sections	Semiconductor nanocrystal probes for human metaphase chromosomes	Xiao, et al. [31]	2004
21	Raman-FISH	Finding out the microbial communities at a single-cell resolution.	Raman-FISH: combining stable isotope Raman spectroscopy and fluorescence in situ hybridization for the single cell analysis of identity and function	Huang, et al. [32]	2007
22	Reverse-FISH	For characterizing of chromosomes and chromosome amplifications in cancer	Fluorescence in situ hybridization with Alu and L1 polymerase chain reaction probes for rapid characterization of human chromosomes in hybrid cell lines.	Lichter, et al. [33]	1990
23	RING-FISH	Identification of individual genes and detection of halo appearance from fluorescence signals at the bacterial cell at periphery	In situ functional gene analysis: recognition of individual genes by fluorescence in situ hybridization	Zwirglmaier, et al. [34]	2005
24	RNA-FISH	Allelic-specific expression in per cell basis	RNA-FISH to analyze allele-specific expression	Braidotti, et al. [35]	2001
25	T-FISH	Mapping of gene loci and looking for specific transcripts in cells	Detection of t(11;18) (q21;q21) in marginal zone lymphoma of mucosa-associated lymphocytic tissue type on paraffin embedded tissue sections by using fluorescence in situ hybridization.	Nomura, et al. [36]	2003

### Increasing trends of FISH technology around the world

**Figure 3:**
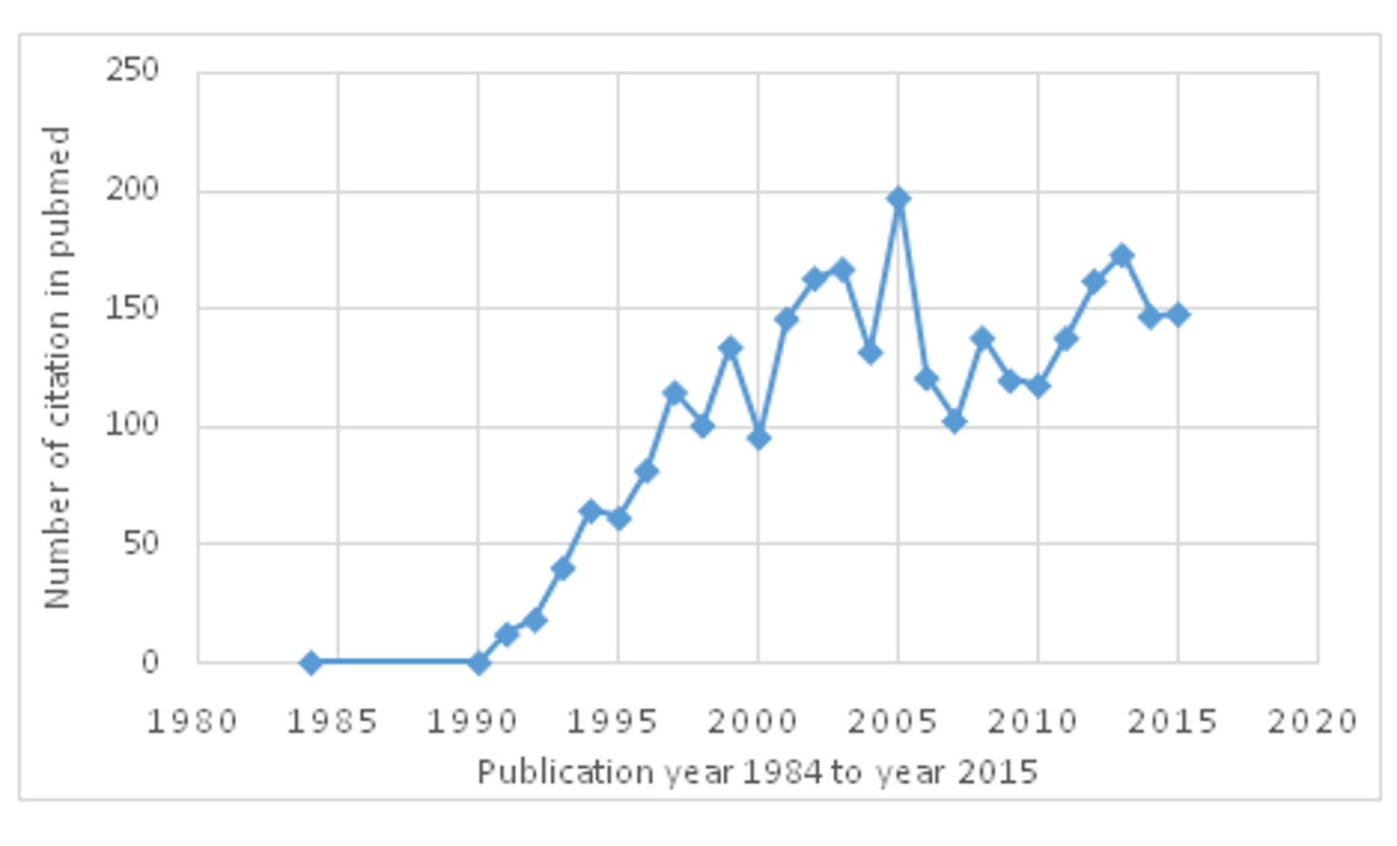
Number of citations in the PubMed database for the keywords ‘ﬂuorescence in situ hybridization'

We carried out a search for citations in the PubMed database using the keywords ‘ﬂuorescence in situ hybridization’, 'genetic anomalies and its correction', and "application of FISH" by publication year. We found that the technique, fluorescence in situ hybridization (FISH), was first introduced in the early 1980's. The FISH assay gained popularity after the 1990s. A new arena of research was started by this application (Figure 3).

### Review of the FISH technology in medical science

Histiocytoid Sweet Syndrome

Histiocytoid Sweet syndrome which is known as acute febrile neutrophilic dermatosis was initially described by Robert Douglas Sweet. It is a dermatological disease with an underlying neutrophilic infiltrate. Sweet syndrome is closely associated with an either or combination of the underlying hematologic myeloid disorder or solid tumor malignancies or inflammatory bowel disease or gastrointestinal tract or upper respiratory tract infections. Here, FISH was performed to determine the presence of BCR/ABL gene fusion. Traditional approaches cannot fulfill the diagnostic criteria of the sweet syndrome. Hence, the FISH was conducted to assess the presence of a chromosomal abnormality in the cutaneous infiltrate of the initial biopsy specimen. Histiocytic Sweet syndrome may indicate leukemia cutis as well [37].

Differential Diagnosis of Pseudomosaicism from True Mosaicism

Interphase FISH in uncultured amniocytes provided a rapid differential diagnosis of pseudomosaicism from true mosaicism. However, it is fetal abnormalities to which true mosaicism for isochromosome 20q has been associated. On uncultured amniocytes, molecular cytogenetic analyses by interphase FISH can be used as a vital tool for a differential diagnosis of pseudomosaicism from true mosaicism in isochromosome 20q detection [38].

Autologous Fat Grafting

Autologous fat grafting has been widely used in many reconstructive surgeries, such as aesthetic skeletal remodeling, breast reconstruction, facial rejuvenation, and facial reconstruction [39]. FISH can be used to detect specific DNA or ribonucleic acid (RNA) sequences within the context of the cell. Here, the technique may help the surgeon to determine the degree of angiogenesis and adipogenesis that arises from the recipient versus grafted cells.

Dedifferentiated Liposarcoma (DDLPS)

For a better prognosis in distinguishing dedifferentiated liposarcoma (DDLPS), it is important to distinguish DDLPS earlier from other high-grade spindle and pleomorphic sarcomas. Mouse double minute 2 homolog (MDM2) amplification and expression are potentially crucial in distinguishing between DDLPS and other undifferentiated high-grade spindle and pleomorphic sarcomas. MDM2 fluorescence in situ hybridization provided excellent data for distinguishing the diseases [40].

Streptococcus Pneumonia

Streptococcus pneumonia can be detected through FISH from blood culture samples. Streptococcus pneumonia (pneumococcus) plays as a major causative agent for bacteremia in both children and adults [41]. However, the FISH procedure without enzymatic treatment can identify S. pneumonia in blood culture specimens. Studies in Bangladesh found that the new technology could be a useful intervention to detect newborn specific disease [42].

Aneuploidies

Studies strongly suggested that the age-related aneuploidies are mainly due to nondisjunction occurring during maternal meiosis [43]. However, the study of first and second polar bodies with the help of one of the cytogenic methods, enable us to detect aneuploidy oocytes in IVF patients. Thus, it can help us to formulate a way to prevent the transfer of embryos resulting from aneuploidy oocytes. It may reduce the chances of an IVF couple to have a child with Down syndrome and other common aneuploidies. With the help of FISH, the detection of chromosome signals in interphase nuclei is possible. Hence, this may be a reliable method for detection of common aneuploidies before the implantation takes place [44].

### Application of FISH in oncology

Chronic Myeloid Leukemia (CML)

The abnormal fusion proteins resulting from chromosomal rearrangements have remarkably contributed in the molecular mechanism of leukemia. The BCR/ABL1 translocation often occurs in chronic myeloid leukemia (CML). The FISH assay is used as the gold standard for detecting these chromosomal translocations, and thus, it can be used as a vital tool in selecting a targeted therapy in different leukemias [45].

Multiple Myelomas (MM)

Multiple myelomas are the heterogeneous malignancy of terminally differentiated B cells. Molecular studies suggested that primary translocations occur in the early stage of MM, followed by a huge number of secondary translocations in the time of tumor progression [46]. Here, FISH is effective for analysis of interphase nuclei and small chromosomal aberrations, which are recognized as the most vigorous genetic test for characterization of cytogenetic abnormalities in MM.

Pulmonary Adenocarcinomas

Anaplastic lymphoma kinase (ALK) rearrangements are related with pulmonary adenocarcinomas. ALK rearrangements are mostly found from the fusion of the echinoderm microtubule-associated protein like 4 (EML4) with ALK at chromosome 2p23 [47]. EML4-ALK gene fusion can be detected through FISH.

Prostate Cancer 

In half of the prostate cancer cases, androgen-regulated TMPRSS2 and E26 transformation-specific (ETS) family members (ERG, RTV1, and ETV4) were detected [48]. Invariably, chromosome rearrangement involves the fusion of TMPRSS2 to the oncogene ETS-related gene (ERG), which leads to the abnormal activation of ERG. In recent years, a four-color FISH assay was used for the detection of either TMPRSS2 or ERG rearrangements.

Breast Carcinomas

In 10% to 20% of human breast carcinomas, overexpression of HER2 is observed [49]. The HER2 protein is an active tyrosine kinase which plays a major role in normal cell growth and differentiation. The HER2 status is crucial for the recommended targeted therapy. At present, FISH assays are used for measuring HER2 overexpression. The FISH assay is regarded as a vital tool for clinical evaluation of the HER2 status.

Renal Mesenchymal Neoplasm

Appropriate identification of renal mesenchymal neoplasms is challenging. Diagnosis can be difficult with needle biopsy; here, immunohistochemistry [38] and in-situ hybridization are used for the accurate diagnosis.

Cholangiocarcinoma (CC)

Cholangiocarcinoma (CC) is regarded as a rare malignancy of the gastrointestinal tract with a poor outcome. Because of the limited entrance to the tumor site, the preoperative diagnosis depends upon the interventional imaging techniques, mainly, the endoscopic retrograde cholangiopancreatography (ERCP) [50]. However, it is also difficult to collect suitable tissue samples. Thus, obtaining genetic information of CC is hampered. Studies showed that the alternative FISH technique on brushing smears can detect numerical and structural abnormalities of four chromosomes in patients with documented extrahepatic CC.

Melanoma

Melanoma is a type of cancer that develops from the pigment-containing cells known as melanocytes. It can be accurately detected through FISH. For the diagnosis, four probes targeting 6p25 (RREB1), 6q23 (MYB), 11q13 (CCND1), and centromere 6 (CEP6) are used. The optimal algorithms for detecting positive FISH results based on these four probes are also established [49].

### Limitation of the review

The present article systematically reviewed the present literature to show the benefits of using FISH. However, meta-analyses should be conducted in future to prove the benefits of FISH over the other conventional techniques.

## Conclusions

In comparing a regular application of clinical diagnosis, the FISH is much more accurate and straightforward, as well as reliable, than the other molecular profiling techniques, e.g., array-based comparative genomic hybridization, single nucleotide polymorphism (SNP), etc. Moreover, FISH gained great recognition as a physical mapping technique to support large-scale mapping and sequencing efforts related to the human genome project. We can visualize and conceptualize genes, chromosomes, transcription, and nucleic acid movements through FISH. FISH also provides reliable biomarker information. In terms of using the FISH technology around the world, we found that the FISH technique has very limited use in developing countries because of unavailability and lack of expert knowledge. Hence, FISH should be the preferred approach in anticipating the complex components of gene expression leading to any disease.
